# Initial central venous pressure could be a prognostic marker for hemodynamic improvement of polymyxin B direct hemoperfusion: a retrospective cohort study

**DOI:** 10.1186/s40560-016-0186-8

**Published:** 2016-10-10

**Authors:** Hiroyuki Yamada, Tatsuo Tsukamoto, Hiromichi Narumiya, Kazumasa Oda, Satoshi Higaki, Ryoji Iizuka, Motoko Yanagita, Masako Deguchi

**Affiliations:** 1 Department of Nephrology, Graduate School of Medicine, Kyoto University, 54 Shogoin-Kawahara-cho, Sakyo-ku, Kyoto, 606-8507 Japan; 2 Department of Metabolism, Nephrology and Rheumatology, Japanese Red Cross Kyoto Daini Hospital, 355-5 Haruobi, Kamigyo-ku, Kyoto, 602-8026 Japan; 3 Department of Emergency, Japanese Red Cross Kyoto Daini Hospital, 355-5 Haruobi, Kamigyo-ku, Kyoto, 602-8026 Japan

**Keywords:** Polymyxin B, Hemoperfusion, Septic shock, Central venous pressure, PMX-DHP

## Abstract

**Background:**

Direct hemoperfusion with polymyxin B-immobilized fiber column (PMX-DHP) could improve the hemodynamic status of septic shock patients. As PMX-DHP is an invasive and costly procedure, it is desirable to estimate the therapeutic effect before performing the therapy. However, it is still unclear when this therapy should be started and what type of sepsis it should be employed for. In this study, we retrospectively examined the clinical effect of patients treated with PMX-DHP by using central venous pressure (CVP).

**Methods:**

Seventy patients who received PMX-DHP for septic shock during the study period were recruited and divided into a low CVP group (*n* = 33, CVP < 12 mmHg) and a high CVP group (*n* = 37, CVP≧12 mmHg). The primary endpoint was vasopressor dependency index at 24 hours after starting PMX-DHP, and the secondary endpoint was the 28-day survival rate. Additionally, we performed a multivariate linear regression analysis on the difference in the vasopressor dependency index.

**Results:**

The vasopressor dependency index significantly improved at 24 h in the low CVP group (0.33 to 0.16 mmHg^−1^; *p* < 0.01) but not in the high CVP group (0.43 to 0.34 mmHg^−1^; *p* = 0.41), and there was a significant difference between the two groups in the index at 24 h (*p* = 0.02). The 28-day survival rate was higher in the low CVP group (79 vs. 43 %; *p* < 0.01). Multivariate linear regression analysis showed that CVP (*p* = 0.04) was independently associated with the difference in the vasopressor dependency index.

**Conclusions:**

Our study indicates that the clinical effect of PMX-DHP for septic shock patients with higher CVP (≧12 mmHg) might be limited and that the initial CVP when performing PMX-DHP could function as an independent prognostic marker for the hemodynamic improvement.

**Electronic supplementary material:**

The online version of this article (doi:10.1186/s40560-016-0186-8) contains supplementary material, which is available to authorized users.

## Background

Both the 2008 and 2012 Surviving Sepsis Campaign Guidelines (SSCG) recommend the rapid infusion of intravenous fluids until a central venous pressure (CVP) of 8–12 mmHg is achieved during initial resuscitation [1, 2]. However, many studies show that excess fluid accumulation is associated with adverse outcomes in critically ill patients [3–5]. In particular, positive fluid balance seems to be harmful for patients whose comorbid burden includes chronic heart failure and/or chronic kidney disease [6, 7]. In order to avoid excess volume expansion in those patients, it is important to carefully monitor intravascular volume by the following parameters: CVP, stroke volume variance, or extravascular lung water.

Direct hemoperfusion with a polymyxin B-immobilized fiber column (PMX-DHP), which can effectively adsorb bacterial endotoxin and lead to an earlier recovery from shock state, was first reported in 1994, and it has been used for the treatment of septic shock in many countries [8–11]. Although many clinical reports, including two randomized control trials, have shown the clinical effect of adapting PMX-DHP for septic shock patients, there is no clear consensus about the effect of the hemoperfusion [9–12]. As PMX-DHP is an invasive and costly procedure, it is desirable to accurately estimate the therapeutic effect before performing the therapy [11]. However, it is still unclear when this therapy should be started and what type of sepsis it should be employed for.

The utility of CVP as a marker of intravascular volume has been questioned for many years [13, 14]. However, we consider that CVP is one of the most widely used hemodynamic parameters because of the promptness of the measurement and the ability to perform it in any hospital facility. Actually, many clinical studies also demonstrated that high CVP was associated with positive fluid balance [1, 5, 15].

In this study, in order to clarify the application of PMX-DHP for septic shock patients, we retrospectively examined the hemodynamic improvement and the mortality of patients treated with PMX-DHP by using CVP values. Moreover, we investigated whether the CVP values at the start of PMX-DHP could function as an independent prognostic factor for the hemodynamic improvement of the hemoperfusion.

## Methods

### Patients

We conducted a retrospective cohort study among all consecutive patients who received PMX-DHP for septic shock between May 2008 and April 2013 in the intensive care unit (ICU), high care unit (HCU), and cardiovascular care unit (CCU) at the Japanese Red Cross Kyoto Daini Hospital and the Kyoto University Hospital in Japan. After initial resuscitation to achieve the early goal directed therapy (EGDT), PMX-DHP was applied along the Japanese health insurance system, as follows [1]: septic shock patients who require vasopressor support because of endotoxin or gram-negative bacteria. The following patients were excluded: (1) those who were under 18 years old, (2) those who were admitted to the ICU, HCU, or CCU for reasons other than sepsis, (3) those who were not given vasopressors when starting PMX-DHP, and (4) those in whose medical records CVP was not sufficiently recorded.

The Ethics Committee of Kyoto University Graduate School and Faculty of Medicine approved the protocol (E2153). This study was retrospective and used only a data bank while employing the highest privacy policy standards. Therefore, the requirement of informed consent was waived.

### Procedures

Vascular access was placed at the femoral or the internal jugular vein. PMX-DHP with PMX-20R (Toray Industries, Tokyo, Japan) was performed for at least 120 min per session once or twice per patient per day for 2 days. The blood flow volume was 80–120 mL/min. The duration of hemoperfusion was decided by the attending physician. The therapy was terminated when the attending physician deemed it appropriate to conclude PMX-DHP for any reason. The anticoagulant used in PMX-DHP was nafamostat mesilate, low molecular weight heparin, or unfractionated heparin. All other cardiovascular management, including cardiac output management, setting of blood pressure goals, and fluid and inotropic therapy, were performed on the basis of SSCG recommendations by the attending physician.

### Definitions and classification

In this study, we classified the patients into two groups: patients with CVP values greater than or equal to (≧) 12 mmHg when starting PMX-DHP were placed in the high CVP group, while the remaining patients whose CVP values were less than (<) 12 mmHg were placed in the low CVP group. CVP was measured using the standard method when starting PMX-DHP and expressed as mmHg, as described previously [16, 17]. This classification is also based on the SSCG recommendations, which suggest that in mechanical ventilation patients, a higher target CVP of 12 mmHg should be achieved [2].

### Data collection

We employed three parameters in order to compare hemodynamic status among the patients in this study: mean arterial pressure (MAP), inotropic score, and vasopressor dependency index, as described in the preceding studies [9, 18]. Namely, the inotropic score was calculated as follows:

$$ \begin{array}{l}\left(\mathrm{dopamine}\ \mathrm{dose}\ \left[\upmu \mathrm{g}/\mathrm{kg}/ \min \right]\right) \times 1 + \\ {}\left(\mathrm{dobutamine}\ \left[\upmu \mathrm{g}/\mathrm{kg}/ \min \right]\right) \times 1 + \left(\mathrm{epinephrine}\ \mathrm{dose}\ \left[\upmu \mathrm{g}/\mathrm{kg}/ \min \right]\right) \times 100 + \\ {}\ \left(n\mathrm{orepinephrine}\ \mathrm{dose}\ \left[\upmu \mathrm{g}/\mathrm{kg}/ \min \right]\right) \times 100 + \left(\mathrm{phenylephrine}\ \mathrm{dose}\ \left[\upmu \mathrm{g}/\mathrm{kg}/ \min \right]\right) \times 100\end{array} $$

And, vasopressor dependency index was calculated as the inotropic score/MAP. The parameters were calculated before the first PMX-DHP, immediately thereafter and 24 h after the first PMX-DHP.

Relevant clinical background, medical history, and clinical data of all patients were collected at appropriate times during the treatment for sepsis. Basic cardiopulmonary data and laboratory data obtained at the time of starting PMX-DHP were considered baseline values. These included age, sex, body mass index, systolic blood pressure, diastolic blood pressure, dopamine infusion rate, noradrenaline infusion rate, inotropic score, vasopressor dependency index, heart rate, central venous pressure, cardiac output, cardiac index, body temperature, arterial pH, lactate, arterial oxygen tension (PaO_2_)/fractional inspired oxygen (FiO_2_) ratio (P/F ratio), positive end-expiratory pressure (PEEP), renal replacement therapy, surgery, hemoglobin, platelet count, C-reactive protein (CRP), total bilirubin, total protein, Acute Physiologic and Chronic Health Evaluation II (APACHE II) score, Sequential Organ Failure Assessment (SOFA) score, time from admission to care units until starting PMX-DHP, duration of PMX-DHP, total fluid dosage from ICU admission until starting PMX-DHP, site of infection, and microorganism types. Cardiac output and cardiac index were measured by an arterial catheter attached to the Flotrac^TM^ pulse counter device (Vigileo^TM^, Edwards Lifesciences, Irvine, CA, USA) or a pulmonary artery catheter attached to Vigilance^TM^ monitor (Vigileo^TM^, Edwards Lifesciences, Irvine, CA, USA).

### Study outcomes

The primary outcome was vasopressor dependency index at 24 h after starting PMX-DHP. The secondary outcome was the 28-day survival rate.

### Statistical analysis

Statistical analysis was performed using JMP for Macintosh version 10.0.2 software (SAS Institute, Tokyo, Japan). Categorical variables are expressed as the number of patients (%) and were analyzed by using the *χ* ^2^ test or Fisher’s exact test. Continuous variables are expressed as means and 95 % confidence intervals (CIs). Comparison of continuous variables between the two groups was conducted with the *t* test or the Mann-Whitney *U* test, according to the distribution of the variables. Evaluation of significance between groups over time points was done by repeated measure ANOVA. As post hoc analysis, the three pair-wise comparisons of the hemodynamic status within a single group among different time points were made using Bonferroni adjustment. Therefore, *p* values less than 0.016 were considered significant only in this comparison. In the other comparisons, statistical significance was defined as *p* values <0.05. Kaplan-Meier curves were constructed for the comparison of the survival rate in the two groups and were tested for difference using the log-rank test.

To ensure the assumption that the CVP at the beginning of PMX-DHP could be an independently prognostic factor for the hemodynamic improvement of the hemoperfusion, we performed a multivariate linear regression analysis that focused on the difference in the vasopressor dependency index before PMX-DHP and 24 h after hemoperfusion. The relationships between the parameter and continuous variables were examined using Pearson’s correlation coefficient, and categorical variables were examined using Spearman’s R test. All variables with *p* values <0.20 in the univariate analysis were included in the multivariate analysis. To ensure that the assumptions for regression analysis were not violated, an analysis of residuals was carried out. Moreover, we performed multivariate Cox regression analysis to assess the covariates that were associated with time to mortality.

## Results

### Patient characteristics

**Fig. 1:**
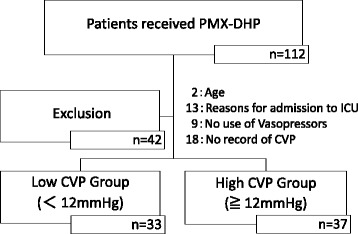
Patient flow diagram for this study. Among 112 patients who received PMX-DHP for septic shock, 70 patients met the requirements of our study but 42 patients were excluded for several reasons as follows: age, 2; reasons for admission to ICU, 13; vasopressors not used, 9; no record of CVP, 18. The 70 patients were the divided into two groups: low central venous pressure (CVP) (CVP <12 mmHg; *n* = 33) and high CVP (CVP ≧12 mmHg; *n* = 37)

Although 112 patients received PMX-DHP for septic shock during the study period, 70 patients met the inclusion criteria, while 42 patients were excluded for a variety of reasons (age, 2; reasons for admission to ICU, 13; vasopressors not used, 9; no record of CVP, 18). Of these 70 patients, the initial CVP of 33 patients when receiving PMX-DHP was <12 mmHg, and the initial CVP of the other 37 patients was ≧12 mmHg, as shown in Fig. 1. The baseline characteristics of the study population are described in Table 1. Although the SOFA score, in particular SOFA liver and SOFA hematological, was significantly higher in the high CVP group than in the low CVP group, the APACHE II score was not significantly different between the two groups. Arterial pH was also significantly lower in the high CVP group. There were no significant differences in the other parameters except CVP. Although cardiac pump dysfunction could have a serious influence on CVP value, there was not a significant difference between the two groups in cardiac output and cardiac index before starting PMX-DHP.

**Table 1:** Baseline characteristics of the patients

	Low CVP group *n* = 33	High CVP group *n* = 37	*p* value
	Median (95 % CI)	Median (95 % CI)	
Age, year	72 (68–76)	67 (64–71)	0.07
Male, *n*(%)	20 (61)	26 (70)	0.46
Body mass index, kg/m^2^	22 (20–23)	23 (21–24)	0.37
Systolic blood pressure, mmHg	99 (90–106)	92 (84–99)	0.22
Diastolic blood pressure, mmHg	50 (47–53)	48 (44–51)	0.30
Mean blood pressure, mmHg	66 (62–70)	62 (59–66)	0.17
Dopamine infusion rate, μg/kg/min	5.0 (3.5–6.6)	5.8 (4.4–7.3)	0.45
Noradrenaline infusion rate, μg/kg/min	0.12 (0.09–0.16)	0.16 (0.12–0.20)	0.20
Inotropic score	20 (15–25)	25 (21–30)	0.14
Vasopressor dependency index, mmHg^−1^	0.33 (0.23–0.45)	0.43 (0.34–0.53)	0.15
Heart rate, bpm	109 (103–116)	115 (109–121)	0.18
CVP, mmHg	8 (7–9)	15(14–16)	<0.01
Cardiac output, L/min	4.6 (3.3–5.8)	5.7 (4.6–5.7)	0.18
Cardiac index, L/min/m^2^	2.8 (2.3–3.4)	3.3 (2.8–3.7)	0.23
Body temperture, °C	36.8 (36.4–37.2)	36.8 (36.4–37.2)	0.92
Arterial pH	7.36 (7.32–7.41)	7.27 (7.23–7.31)	<0.01
Lactate, mmol/L	3.9 (2.6–5.3)	4.0 (2.9–5.2)	0.94
P/F ratio	220 (181–258)	168 (131–206)	0.06
PEEP, cmH_2_O	8 (6–10)	9 (7–11)	0.32
Renal replacement therapy, *n*(%)	13 (39)	23 (62)	0.06
Surgery, *n*(%)	21 (64)	17 (46)	0.16
Hemoglobin, g/dL	10.4 (9.7–11.1)	10.3 (9.6–11.0)	0.78
Platelet count, ×10^9^/L	111 (87–134)	81 (59–104)	0.08
CRP, mg/dL	17.7 (11.4–24.0)	17.6 (11.6–23.5)	0.97
Total Bilirubin, mg/dL	1.7 (0.2–3.1)	3.6 (2.2–5.0)	0.06
Total Protein, mg/dL	4.9 (4.5–5.3)	4.6 (4.2–4.9)	0.17
APACHE II score	26 (25–28)	28 (26–30)	0.23
SOFA score	12 (11–13)	14 (13–15)	0.01
SOFA cardiovascular	3.5 (3.3–3.7)	3.7 (3.5–3.9)	0.29
SOFA renal	1.9 (1.4–2.4)	2.3 (1.8–2.8)	0.22
SOFA hematological	1.6 (1.2–2.0)	2.1 (1.8–2.5)	0.05
SOFA respiratory	2.4 (1.9–2.8)	2.8 (2.4–3.2)	0.15
SOFA liver	0.8 (0.4–1.2)	1.4 (1.0–1.7)	0.04
SOFA central nerve system	2.0 (1.6–2.5)	2.0 (1.5–2.4)	0.83
Time from ICU admission until starting PMX-DHP, min	441 (98–802)	829 (498–1159)	0.11
PMX-DHP duration, min	354 (249–458)	366 (263–469)	0.97
Total fluid dosage from ICU admission until starting PMX-DHP, ml	2970 (1200–4760)	3212 (1223–5203)	0.86

*APACHE* Acute Physiologic and Chronic Health Evaluation, *CRP* c-reactive protein, *CVP* central venous pressure, *ICU* intensive care unit, *PEEP* positive end-expiratory pressure, *P/F ratio* arterial oxygen tension/fractional inspired oxygen ratio *PMX-DHP* direct hemoperfusion with polymyxin B-immobilized fiber column, *SOFA* Sequential Organ Failure Assessment

Table 2 shows the site of infection and microorganism types in both groups. There were also no significant differences between them.

**Table 2:** Isolated microorganisms by treatment group

	Low CVP group *n* = 33	High CVP group *n* = 37
Site of infection		
Abdomen	13	15
Lung	6	13
Urinary tract	7	2
Skin	2	1
Blood stream	0	1
Others	5	5
Microorganism type		
Escherichia coli	7	5
Staphylococcus species	2	5
Streptococcus species	1	4
Enterococcus species	1	2
Pseudomonas species	1	2
Bacteroides species	1	1
Klebsiella species	2	0
Serratia species	1	1
Acinetobacter species	1	0
Citrobacter species	0	1
Clostridium species	1	0
Morallexa species	0	1
Stenotrophomonas species	0	1

### Primary outcome

**Fig. 2:**
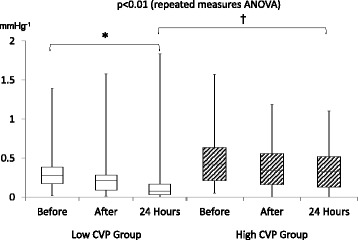
Changes in vasopressor dependency index. Each *box plot* indicates medians, 25th and 75th percentiles, and whisker caps indicate 5th and 95th percentiles. *White box plots* indicate the low CVP group, and the *diagonal-lined box* indicates the high CVP group. An *asterisk* indicates a significant difference with *p* < 0.01, and a *dagger* indicates a significant difference with *p* < 0.05. There was a significant difference between the two groups in the repeated measure ANOVA. The vasopressor dependency index also significantly improved at 24 h in the low CVP group (0.28 to 0.16 mmHg^−1^; *p* < 0.01) but not in the high CVP group (0.43 to 0.34 mmHg^−1^; *p* = 0.10). Additionally, there was a significant difference in the index at 24 h between the two groups (*p* < 0.05)

**Fig. 3:**
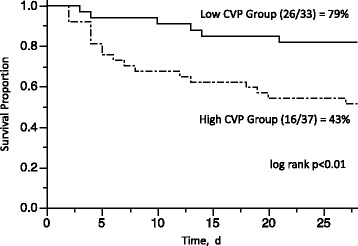
Survival rate of each group by Kaplan-Meier analysis. Although patients in both groups were similarly treated with PMX-DHP, patients in the low CVP group showed a better survival rate in this study

Repeated measures ANOVA for the vasopressor dependency index revealed that the index in the low CVP group improved more significantly than in the high CVP group (for each *p* < 0.01) (Fig. 2). As for post hoc analysis, the vasopressor dependency index decreased significantly at 24 h (0.16 mmHg^−1^; 95 % CI, 0.05–0.28; *p* < 0.01) but not after PMX-DHP (0.24 mmHg^−1^; 95 % CI, 0.15–0.34; *p* = 0.14) in the low CVP group whereas the decrease was observed neither after PMX-DHP (0.39 mmHg^−1^; 95 % CI, 0.30–0.49; *p* = 1.00) nor at 24 h (0.34 mmHg^−1^; 95 % CI, 0.25–0.44; *p* = 0.41) in the high CVP group. Additionally, we could observe a significant difference in the index at 24 h between the two groups (*p* < 0.05) (Fig. 3). Thus, PMX-DHP appeared to be more effective for the hemodynamic status in the low CVP group than in the high CVP group.

### Secondary outcome

The survival rates of both groups after 28 days were analyzed by the Kaplan-Meier method (Fig. 3). The survival rate was significantly higher in the low CVP group than in the high CVP group (*p* < 0.01), as determined by log-rank test.

### Regression analysis

Correlation analyses were performed to identify factors associated with the difference in the vasopressor dependency index before and 24 h after PMX-DHP (Additional file 1: Table S1). In the univariate regression analysis, age statistically significantly correlated with the difference (*p* = 0.03). Subsequent multivariate linear regression confirmed CVP (*p* = 0.04) and age (*p* = 0.03) as independent prognosis factors regarding the hemodynamic improvement of PMX-DHP (Table 3). This also indicates that the higher the CVP at the start of hemoperfusion is, the less it improves the hemodynamic state.

**Table 3:** Results of multivariate regression analysis for the difference in the vasopressor dependency index between before and 24 h after PMX-DHP

Selected variables	Regression coefficient (β)	95 % CI	Partial correlations	*p* value	*r* ^2^
Age	−0.2972	−0.0168 − −0.0009	−0.0089	0.03	0.180
CVP	−0.3155	−0.0348 − −0.0007	−0.0178	0.04	
SOFA	0.1530	−0.0166 − 0.0497	0.0165	0.32	
Arterial pH	−0.2871	−1.2656 − −0.0171	−0.6242	0.06	

*CVP* central venous pressure, *PMX*-*DHP* direct hemoperfusion with polymyxin B-immobilized fiber column, *SOFA* Sequential Organ Failure Assessment

## Discussion

In this study, we observed the association between CVP and the hemodynamic improvement with PMX-DHP. Our results yielded two interesting findings. First, the hemodynamic status of patients with higher CVP did not improve significantly by PMX-DHP. In other words, our retrospective results did not support the guideline’s recommendations, which suggested that septic shock patients with mechanical ventilation should achieve a higher target of CVP 12 to 15 mmHg^2^. Second, the initial CVP when performing PMX-DHP could function as an independent prognostic factor for the hemodynamic improvement of the therapy. To the best of our knowledge, this is the first study that investigated this particular association and prognosis.

Although CVP is one of the most popular hemodynamic parameters, we cannot deny that CVP values may not reflect intravascular volume accurately. In fact, recent reviews reported that the CVP value is mainly determined by two factors: cardiac pump function and venous return function [17, 19, 20]. In terms of cardiac function, a high CVP indicates a decrease in contractility, diastolic dysfunction, valvular disease, and cardiomyopathy in these patients, although in our study, there was not a significant difference in cardiac output and cardiac index [19, 20]. On the other hand, venous return is determined by the gradient between CVP and the mean circulatory filling pressure (MCFP), as shown in the formula below:

$$ \mathrm{venous}\ \mathrm{return} = \left(\mathrm{MCFP} - \mathrm{C}\mathrm{V}\mathrm{P}\right)/\mathrm{venous}\ \mathrm{resistance} $$

[19] MCFP is the pressure in the vasculature when the heart is stopped (zero flow) and the pressures in all segments of the circulatory system have equalized [21, 22]. Thus, an increase in CVP values leads to the decrease in venous return [19–22]. Because PMX-DHP does not directly affect these pressures and cardiac function, it is difficult for the hemoperfusion to improve the hemodynamic status for septic shock patients with high CVP.

Actually, in this study, we observed that the patients in the high CVP group suffered from hemodynamic impairment due to high CVP. The proportion of patients who received renal replacement therapy was non-significantly larger in the high CVP group, which suggests that many of the attending physicians might think the intravascular volume in the high CVP group patients is too large. Additionally, the P/F ratio and PEEP were also non-significantly lower in the high CVP group, which indicated that some of the patients had a high intrathoracic pressure. Hence, we consider that high CVP group patients did not have adequate venous return because excess fluid therapy or high intrathoracic pressure reduces the gradient between MCFP and CVP. On the other hand, the significant improvement in the low CVP group could be because they genuinely received the clinical effect of PMX-DHP. Generally, PMX-DHP can reduce plasma cytokine levels by absorbing endotoxin, immune cells, and anandamide [23–25]. These physiological and pathological responses could be equivalent in both groups in our study. However, the harmful effect of high CVP at the start of PMX-DHP differentiated the clinical effect of both groups. In other words, fluid toxicity or increase in intrathoracic pressure might be deleterious beyond the beneficial effect of PMX-DHP in patients with high CVP in our study.

Previous studies have reported that early initiation of PMX-DHP reduced the catecholamine requirement and that early improvement in inotropic score and vasopressor dependency index after PMX-DHP might be a prognostic factor [18, 23, 26]. In our study, the patients in the low CVP group who received PMX-DHP earlier also tended to show a decrease in their vasopressor dependency index. Meanwhile, in terms of the time between ICU admission and starting PMX-DHP, there was neither a statistical difference between the two groups nor a significant association with hemodynamic improvement in our study. However, because the sample size of our study was not large enough to demonstrate the association, our results should be viewed with this limitation in mind. In addition, although we performed PMX-DHP for around 6 h in both groups, a recent study has indicated that a longer duration of PMX-DHP therapy can be expected to improve the hemodynamics and pulmonary oxygenation capacity of patients with severe sepsis/septic shock [27]. Thus, longer operation of PMX-DHP might contribute to improve the outcome of patients with low CVP.

Other limitations of our study need to be acknowledged. First, we could not show the data on intrinsic PEEP, so-called auto-PEEP, which might have a direct influence on the CVP values in the high CVP group. However, we consider that it may not fundamentally change our conclusion. Auto-PEEP-induced hypotension is not a result of hyper-inflammatory response to sepsis, but it is rather a patient-ventilator interaction. Thus, it is obvious that PMX-DHP is less effective for the high CVP patients with auto-PEEP-induced hypotension. Additionally, this clinical study is not for acute exacerbation of chronic obstructive pulmonary disease, bronchial asthma, or acute respiratory distress syndrome, and it only evaluated patients with septic shock. Thus, we consider that there were not a large proportion of the study patients with auto-PEEP in our study. Second, the previous studies reported that high CVP was associated with a poor prognosis [28, 29]. Indeed, high CVP might have a negative influence on the cardiac function during the treatment in patients with high CVP group, although cardiac output and index were not significantly different in both groups at the beginning of the hemoperfusion [29]. Therefore, regardless of the effect of PMX-DHP, there may be a possibility of observing the clinical course of patients with a poor prognosis. Third, this study was not a randomized controlled trial, and we cannot rule out the possibility of selection bias, especially referral bias and Neyman bias. Fourth, perhaps we could not extract the patients whose conditions changed rapidly or whose case was extremely severe because our study patients had enough time to receive the hemoperfusion. Fifth, vasopressors were regulated by local physicians, depending on the patient’s condition. Therefore, the protocol for titrating the vasopressors was different among the attending physicians. Further study is required to clarify these unsolved issues.

## Conclusions

Our study indicated that the effect of PMX-DHP for septic shock patients with higher CVP (≧12 mmHg) may be limited and that the initial CVP in performing PMX-DHP could be an independent prognostic marker for hemodynamic improvement. Further study is required to clarify the mechanisms of PMX-DHP that affect sepsis treatment.

## Additional file

Additional file 1:
**Table S1.** Results of univariate regression analysis for the difference in the vasopressor dependency index between before and 24 h after PMX-DHP. (XLSX 12 kb)

## Abbreviations

APACHE: Acute Physiologic and Chronic Health Evaluation
CCU: Cardiovascular care unit
CRP: C-reactive protein
CVP: Central venous pressure
EGDT: Early goal directed therapy
ER: Emergency room
HCU: High care unit
ICU: Intensive care unit
MAP: Mean arterial pressure
MCFP: Mean circulatory filling pressure
P/F ratio: Arterial oxygen tension/fractional inspired oxygen ratio
PEEP: Positive end-expiratory pressure
PMX-DHP: Direct hemoperfusion with polymyxin B-immobilized fiber column
SOFA: Sequential Organ Failure Assessment
SSCG: Surviving Sepsis Campaign Guidelines
